# How many cases are required to achieving early proficiency in purely off-clamp robot-assisted partial nephrectomy?

**DOI:** 10.3389/fsurg.2023.1309522

**Published:** 2024-01-03

**Authors:** Guoling Zhang, Bowen Wang, Hua Liu, Guang Jia, Boju Tao, Haoxun Zhang, Chunyang Wang

**Affiliations:** Department of Urology, The First Affiliated Hospital of Harbin Medical University, Harbin, China

**Keywords:** kidney cancer, robot-assisted partial nephrectomy, robotic surgery, off-clamp, learning curve, cumulative sum analysis, perioperative outcomes, warm ischemia time

## Abstract

**Background and purpose:**

Off-clamp robot-assisted partial nephrectomy (Offc-RAPN) is a technically challenging procedure that can effectively avoid renal ischemia owing to the absence of hilar vessel preparation and clamping. However, data on the learning curve (LC) for this technique are limited. The purpose of this study was to assess the LC of Offc-RAPN and compare the perioperative outcomes between different learning phases.

**Methods:**

This retrospective study included 50 consecutive patients who underwent purely Offc-RAPN between January 2022 and April 2023. Multidimensional cumulative sum (CUSUM) analysis method was used to assess LC. Spearman's correlation and LOWESS analysis were performed to analyze the continuous variables of perioperative outcomes. Baseline characteristics and perioperative outcomes were compared using *χ*^2^-test, *t*-test and *U*-test.

**Results:**

CUSUM analysis identified two LC phases: phase I (the first 24 cases) and phase II (the subsequent 26 cases). Phase II showed significant reductions in mean operative time (133.5 vs. 115.31 min; *p* = 0.04), mean console time (103.21 vs*.* 81.27 min; *p* = 0.01), and mean postoperative length of stay (5.33 vs*.* 4.30 days; *p* = 0.04) compared to phase I. However, no significant differences were observed in other perioperative outcomes or baseline characteristics between the two LC phases.

**Conclusions:**

Offc-RAPN performed by a surgeon with experience in laparoscopic and robotic surgeries achieved early proficiency in 24 cases. Moreover, Offc-RAPN alone is safe and feasible even in the initial phase of the LC for an experienced surgeon.

## 1. Introduction

The treatments for localized renal cell carcinoma include surveillance, ablation therapy, radical nephrectomy, and partial nephrectomy, among which partial nephrectomy has become the preferred approach for localized T1 stage treatment whenever technically feasible (1, 2). Robot-assisted partial nephrectomy (RAPN) has become increasingly popular in the past decade because of its advantages in tumor resection and renal reconstruction (3).

On-clamp RAPN (Onc-RAPN) temporarily clamps hilar vessels facilitating tumor excision and renorrhaphy, yet it carries the risk of renal injury (4). To minimize renal impairment, off-clamp RAPN (Offc-RAPN) has been shown to be a safe and feasible alternative technique (5–7) However, Offc-RAPN remains a complex surgical technique potentially associated with an increased risk of intraoperative hemorrhage (8–10). The technical complexity presents a significant challenge for surgeons without prior experience in Offc-RAPN.

The learning curve (LC) reflects the progression of skill acquisition and understanding of novel surgical technique (11). Although previous studies have reported the LC of RAPN (12, 13), research has been scarce regarding the learning process of purely Offc-RAPN. Assessing the LC of Offc-RAPN is crucial to ensure patient safety and optimize surgical outcomes throughout the learning period.

Therefore, this study was designed to assess the LC of Offc-RAPN and compare the perioperative outcomes between the different phases of the learning performed by a single surgeon in a consecutive series of patients.

## 2. Materials and methods

### 2.1. Patients

Prospectively maintained and ethics commitment-approved renal tumor databases were analyzed. Out of the 127 RAPN patients, 50 consecutive patients who underwent purely Offc-RAPN at our center between January 2022 and April 2023 were included in this study. All procedures were performed by the same surgeon (C. Wang) using the da Vinci Xi Surgical System (Intuitive Surgical, Sunnyvale, CA, USA). The surgeon had performed >100 robotic surgeries and >500 laparoscopic surgeries prior to this series.

**Figure 1:**
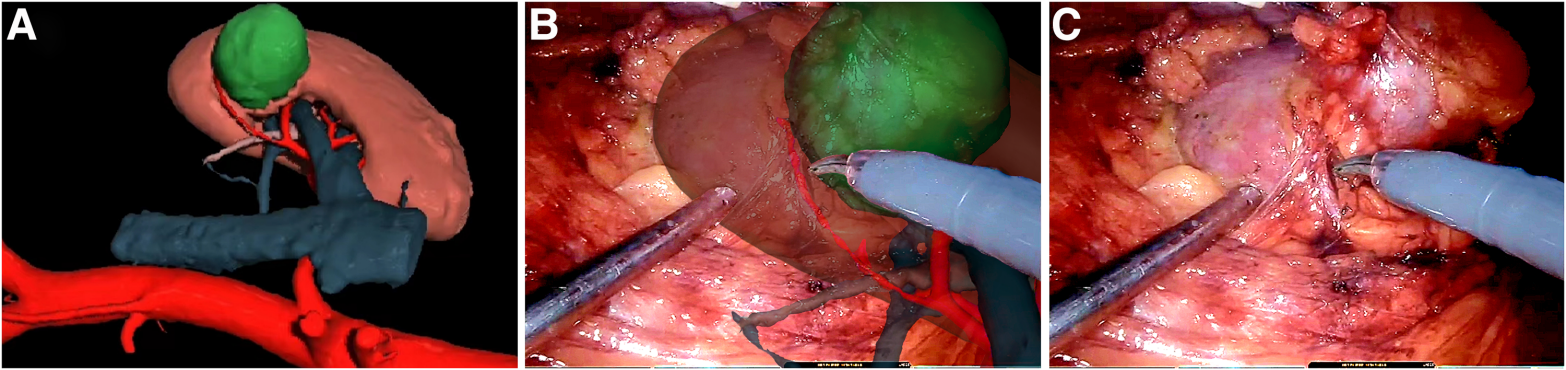
(**A**) Three-dimensional virtual image of the tumor and renal parenchyma; (**B**) overlapping of virtual images enhances vasculature and the ureter in the right kidney; (**C**) dissecting tumor and tumor-feeding vessel from the right kidney.

**Figure 2:**
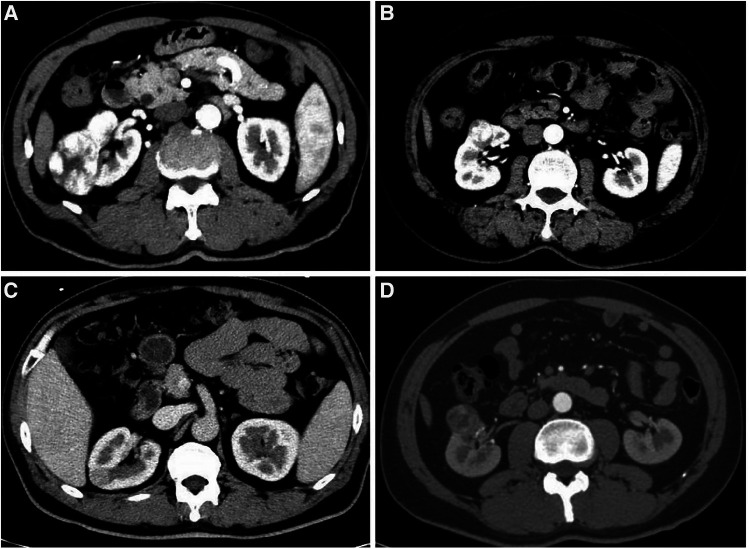
The images represent actual cases of offc-RAPN. (**A**) preoperative CT depicting the largest diameter of a large and exophytic rate >50% tumor within the renal parenchyma <4 cm; (**B**) preoperative CT showing the largest diameter of an exophytic rate <50% tumor within the renal parenchyma <2 cm; (**C**) preoperative CT revealing the largest diameter of an endophytic tumor <2 cm; (**D**) preoperative CT presenting the largest diameter of an exophytic rate >50% and hilar tumor <4 cm.

Contrast-enhanced computed tomography with 1-mm slices was performed to adequately assess the degree of adherent perinephric fat (APF) and the anatomical characteristics of the tumor and renal vasculature. For completely intraparenchymal or located hilar tumors, additional three-dimensional reconstruction would be implemented (Figure 1). Important criteria for purely Offc-RAPN included (Figure 2): (1) exophytic rate ≥50% and cT1 renal tumors, the largest diameter within the renal parenchyma ≤4 cm; (2) exophytic rate <50% renal tumor, the largest diameter within the renal parenchyma ≤2 cm; (3) endophytic renal tumors, the largest diameter ≤2 cm (4) hilar tumors, the largest diameter ≤4 cm.

### 2.2. Data collection and definition

Baseline demographics included sex, age, American Society of Anesthesiologists physical status score, body mass index (BMI), tumor size, R.E.N.A.L. nephrometry score, and Mayo Adhesive Probability (MAP) score. Preoperative variables included serum creatinine (Scr), estimated glomerular filtration rate (eGFR), and hemoglobin (Hb) levels. Perioperative parameters comprised operative time (OT), console time, complications, estimated blood loss (EBL), postoperative length of stay (PLOS), postoperative Scr, eGFR, and Hb levels. Pathological outcomes, including histopathology and surgical margin status, were also evaluated. The eGFR was calculated using the Chronic Kidney Disease Epidemiology Collaboration formula (14). Perioperative complications were reported according to the Clavien-Dindo classification (15). Trifecta achievement was defined as meeting three distinct criteria: negative surgical margin, reduction in eGFR < 10%, and Clavien-Dindo grade <2 complications. The time from the skin initial incision to the completion of wound closure was defined as OT, whereas the console time was defined as the period from the operation of robot to detachment during the procedure.

### 2.3. Surgical technique

A Foley catheter was inserted into patients after induction of general anesthesia, positioned in the flank at a 60-degree extension, and fastened securely to the surgical bed. All pressure points on the patient were placed on padding. The selection of the surgical approach, whether retroperitoneal or transperitoneal with a three-arm configuration plus one assistant port technique, was determined based on tumor characteristics, patient's surgical history, and surgeon's preference. Generally, the retroperitoneal approach is preferred.

After sweeping the extraperitoneal fat, Gerota's fascia was incised and the perirenal fat was carefully dissected from the kidney to expose the tumor and the adjacent renal parenchyma. The kidney was adequately mobilized when necessary to facilitate an optimal surgical space. The aspiration of excessive free fat from the assistant port within a safe area requires a cutting and aspiration technique. Hilum identification and preparation had the potential for vascular complications during dissection and exposure of the hilar vessels, which was carried out only in the first 7 cases.

Intraoperative robotic ultrasonography was used to evaluate the tumor border and depth. Subsequently, monopolar electrocautery scored the marker sulcus around the tumor edge. A radial nephrotomy incision was made perpendicularly to the marked sulcus using scissors. The relatively avascular plane was identified and circumferentially advanced at its edge using a combination of blunt and sharp dissections. Reverse traction was exerted using a suction device and robotic bipolar forceps to stabilize and expose the relatively avascular plane. Depending on the visibility of the tumor capsule, the tumor and capsule were entirely resected from the resection bed, or the tumor was resected along with approximately 5 mm of surrounding healthy tissue. Pneumoperitoneum pressure was temporarily increased to 18 mmHg during tumor resection.

Most hemostasis was effectively accomplished using bipolar electrocautery (75 W) alone during tumor resection and renorrhaphy. However, for larger (visible) tumor-feeding vessels, pre-hemostasis was performed using Hem-o-lock clips (Sinolinks, Chang Zhou, China). If sudden spurting bleeding occurs, the suction cannula promptly compresses the bleeder and optimizes the visibility of the surgical field (approximately one-quarter of the suction flow rate). Subsequently, robotic bipolar forceps occluded and cauterized the bleeder. When bleeding persisted, the bleeding was temporarily occluded using robotic bipolar forceps and ligated with 2-0 resorbable polyglactin sutures (Vicryl; Ethicon, Somerville, NY, USA) after the tumor resection. The remaining bleeders within the resection bed were controlled by cauterization or Hem-o-lock clips (Sinolinks). The collecting system injuries were adequately sutured using a 3-0 V-Loc suture (Covidien, Mansfield, MA, USA) before renal reconstruction.

The renal defect edges were secured using a 2-0 or 3-0 V-Loc suture (Covidien), preloaded with a hem-o-lock clip (Sinolinks) at the tail end. For defects of ≤2 cm, a single-layer running renorrhaphy was performed using the 2-0 V-Loc suture, placing another Hem-o-lock clip (Sinolinks) on the loose end. The sliding clip technique (16) was used to adjust the renorrhaphy bed tension. For defects exceeding 2 cm, the closure of the inner defect was achieved using a 3-0 V-Loc suture (Covidien) in a running fashion. The renorrhaphy bed was closed using 2-0 V-Loc sutures (Covidien), and the sutures were tightened sequentially using the sliding clip technique. If necessary, additional sutures were used to rectify the existing defects in the renorrhaphy bed. When renorrhaphy was completed, the renorrhaphy bed and urine were inspected for at least 5 min with a 5-mmHg insufflation pressure to ensure hemostasis. Finally, a drainage tube was placed adjacent to the bed.

### 2.4. Postoperative management

Important immediate postoperative considerations included the monitoring of vital signs as well as the contents and output of the drainage tube and Foley catheter. Patients were instructed to remain on three-day bedrest. During the third PLOS, a laboratory workup was conducted to evaluate Hb, eGFR, and Scr. If no indication of active bleeding was observed in the laboratory results, the Foley catheter was removed after ambulation, and the drainage tube was removed when the daily drainage was <20 ml.

### 2.5. Statistical analysis

Multidimensional cumulative summation (CUSUM) analysis was used to estimate quantitatively the learning curve (17). Four key assessment indicators, specifically, OT, PLOS, EBL, and complications, were quantized as *α*_1_, *α*_2_, *α*_3_, and *α*_4_, respectively. The criteria for achieving surgical competence were defined as achieving target values for each parameter: OT, EBL, PLOS < mean value, and Clavien-Dindo grade <2 complications. The quantized value of these assessment indicators was calculated using equation S_i_ = *α*_1_ + *α*_2_ + *α*_3_ + *α*_4_ and *α*_i _= X_i_–X_0_. Here, X_0_ represented the parameter's failure rate. If a parameter achieved its target value, X_i_ was set to 0; otherwise, it was assigned 1. Subsequently, the S_i_ values were summed according to the equation CUSUM = ∑S_i_. To visualize the learning curve, we plotted the cumulative values of these indicators for each case and conducted polynomial curve fitting. A successful breakthrough in the learning curve was indicated when the slope transitioned from positive to negative.

Quantitative data were presented as means and standard deviations, while frequencies and percentages were used for qualitative data. Group differences were analyzed using *χ*^2^-test, *U*-test, and *t*-test. The relationship between the number of cases and the continuous variables of perioperative outcomes was assessed using Spearman's correlation and LOWESS analysis. Statistical significance was determined by considering *p*-values <0.05. Origin 2021 software (Origin Lab Corporation, Northampton, MA, USA) was used for the statistical analyses and graphing.

## 3. Results

### 3.1. Patient demographic and preoperative outcomes

The baseline demographics of the patients are shown in Table 1. Between January 2022 and April 2023, 50 patients underwent successful Offc-RAPN. Of these patients, 20 (40%) were females; their mean age was 57.64 year, with a mean BMI 25.72 kg/m^2^. The mean R.E.N.A.L. nephrometry score was 5.62.

**Table 1:** Baseline demographic and preoperative variables of patients.

Variables	Total (*n* = 50)	Phase I (*n* = 24)	Phase II (*n* = 26)	*p*-value
Age^b^ (y)	57.64 (10.59)	57.83 (10.42)	57.46 (10.96)	0.92
Gender^b^, *n* (%)				
Female	20 (40)	9 (37.5)	11 (48.3)	0.73
BMI^c^ (kg/m^2^), mean (SD)	25.72 (3.81)	25.6 (3.2)	25.83 (4.11)	0.71
ASA score^b^, *n* (%)				0.18
1–2	34 (68)	6 (25)	11 (42.3)	0.17
3	16 (32)	18 (75)	15 (51.7)	
Tumor size^c^ (cm), mean (SD)	3.13 (1.69)	2.9 (1.00)	3.33 (2.13)	0.74
Tumor size of renal parenchyma^a^ (cm), mean (SD)	2.32 (0.80)	2.22 (0.87)	2.41 (0.73)	0.23
RENAL score^c^, mean (SD)	5.62 (1.38)	5.46 (1.22)	5.77 (1.39)	0.39
RENAL score^b^, *n* (%)				0.50
4–6	41 (82)	20 (83.3)	21 (80.8)	
7–9	8 (16)	4 (16.7)	4 (15.4)	
10–12	1 (2)	0	1 (3.8)	
MAP score^b^, *n* (%)				0.55
0–2	36 (72)	19 (79.2)	17 (65.4)	
3–4	11 (22)	4 (16.7)	7 (26.9)	
5	3 (6)	1 (4.2)	2 (7.7)	
Preoperative Scr^c^ (µmol/L), mean (SD)	73.52 (24.20)	76.24 (29.08)	70.99 (18.64)	0.44
Preoperative eGFR^c^ (ml/min), mean (SD)	96.93 (21.75)	96.01 (22.25)	97.77 (21.68)	0.61
Preoperative Hb^a^ (g/L), mean (SD)	142.5 (16.3)	141.58 (16.45)	143.35 (16.43)	0.71

BMI, body mass index; ASA, American society of anesthesiologists; MAP, mayo adhesive probability; Scr, serum creatinine; eGFR, estimated glomerular filtration rate; Hb, hemoglobin; SD, standard deviations.

^a^ *t*-test.

^b^ *χ*^2^-test.

^c^ *U*-test.

### 3.2. Perioperative outcomes

Perioperative outcomes are listed in Table 2. Three cases (6%) with Clavien-Dindo grade >2 complications needed blood transfusion. The mean EBL was 119.2 ± 185.18 ml, and the Hb change was −15.26 ± 14.62 g/L. Five (8%) patients experienced eGFR declines exceeding 10%. The rate of trifecta achievement rate was 86% (43/50). The mean console time was 91.8 ± 32.1 min, and the OT was 124.04 ± 31.55 min. The mean PLOS was 4.8 ± 1.22 days. None of the patients had a positive surgical margin, and 38 (76%) were diagnosed with malignant pathology.

**Table 2:** Pathologic and perioperative outcomes.

Variables	Total (*n* = 50)	Phase I (*n* = 24)	Phase II (*n* = 26)	*p*-value
Postoperative Scr^c^ (µmol/L), mean (SD)	76.81 (23.03)	80.52 (28.15)	73.40 (16.88)	0.22
Scr change^c^ (µmol/L), mean (SD)	3.30 (7.38)	4.28 (5.75)	2.40 (8.64)	0.44
Postoperative eGFR^c^ (ml/min), mean (SD)	94.33 (20.56)	92.17 (19.79)	96.33 (21.44)	0.39
eGFR change^c^ (%), mean (SD)	−2.88 (6.69)	−3.38 (5.75)	−2.42 (7.55)	0.76
Postoperative Hb^a^ (g/L), mean (SD)	127.68 (20.56)	128.21 (18.85)	127.19 (22.38)	0.86
Hb change^c^ (g/L), mean (SD)	−15.26 (14.62)	−13.37 (11.01)	−17 (17.35)	0.44
EBL^c^ (ml), mean (SD)	119.2 (185.18)	117.92 (164.74)	120.38 (205.53)	0.78
PLOS^c^ (d), mean (SD)	4.8 (1.22)	5.33 (1.46)	4.30 (0.68)	0.04
OT^a^ (min), mean (SD)	124.04 (31.55)	133.5 (35.56)	115.31 (24.95)	0.04
Console time^c^ (min), mean (SD)	91.8 (32.1)	103.21 (33.76)	81.27 (27.04)	0.01
Trifecta achievement^c^, *n* (%)	43 (86)	22 (91.7)	21 (80.8)	0.27
Clavien-Dindo grade complications^b^, *n* (%)				0.60
<2	50 (94.3)	23 (95.8)	27 (93.1)	
≥2	3 (5.7)	1 (4.2)	2 (6.9)	
Positive Surgical Margins^b^, *n* (%)	0	0	0	1.00
Histology^b^, *n* (%)				0.41
Benign	12 (24)	7 (29.2)	5 (19.2)	
Malignant	38 (76)	17 (70.8)	21 (80.8)	

EBL, estimated blood loss; PLOS, postoperative length of hospital stay; OT, operative time; SD, standard deviations.

^a^ *t*-test.

^b^ *χ*^2^–test.

^c^ *U*-test.

**Figure 3:**
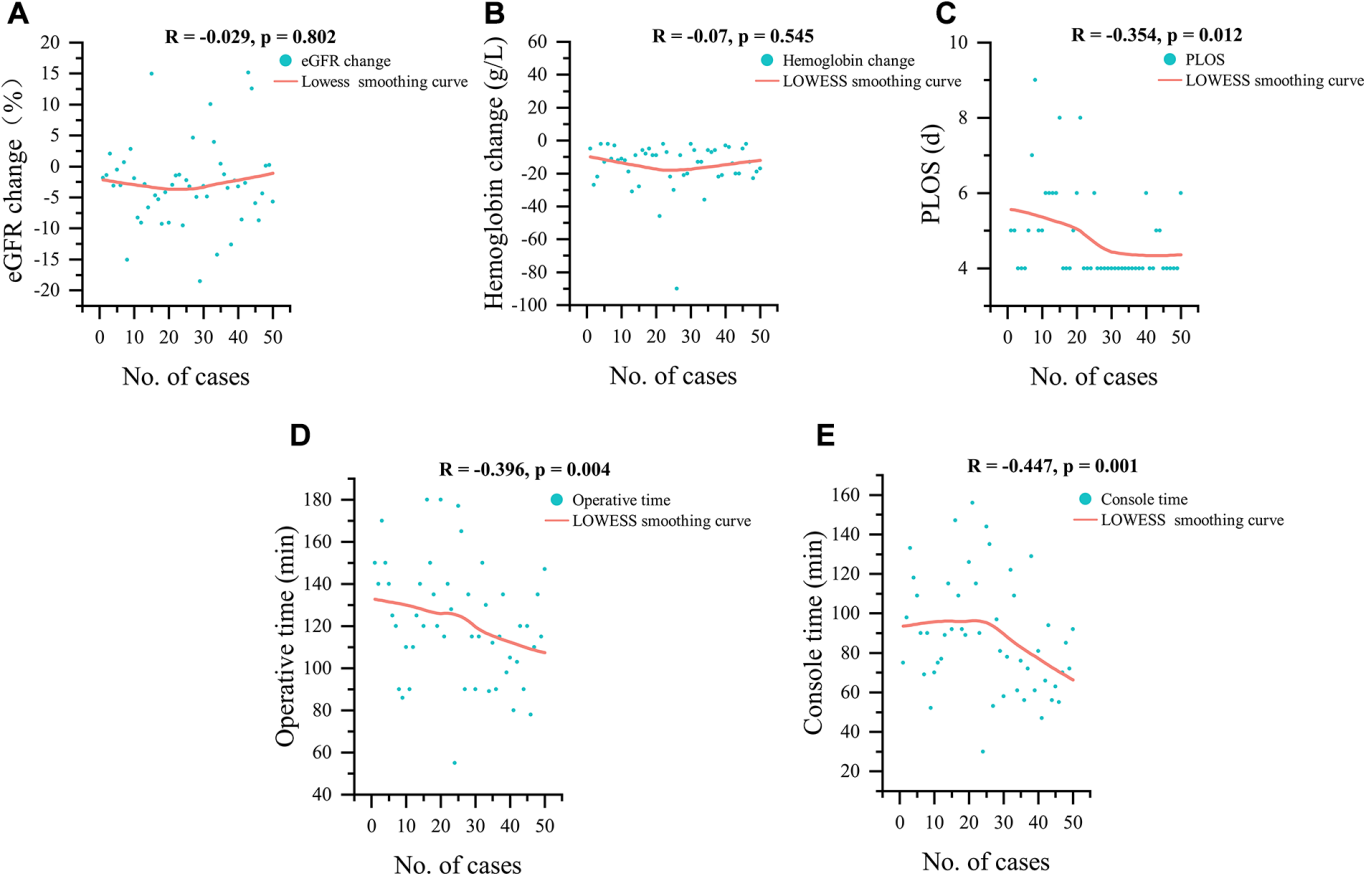
LOWESS smoothing curves for 50 cases, (**A**) eGFR change, (**B**) hemoglobin change, (**C**) postoperative length of stay, (**D**) operative time and (**E**) console time; eGFR, estimated glomerular filtration rate; PLOS, postoperative length of stay.

Figure 3 illustrates the relationship between the number of cases and the continuous variables of perioperative outcomes. A downward trend was observed with increased surgical experience for PLOS (*R* = −0.354, *p* = 0.012), OT (*R* = −0.396, *p* = 0.004), and console time (*R* = −0.447, *p* = 0.001;). However, no significant trends in Hb change (*R* = −0.07, *p* = 0.545), eGFR change (*R*  = −0.029, *p* = 0.802) were detected.

### 3.3. Learning curve analysis with CUSUM

**Figure 4:**
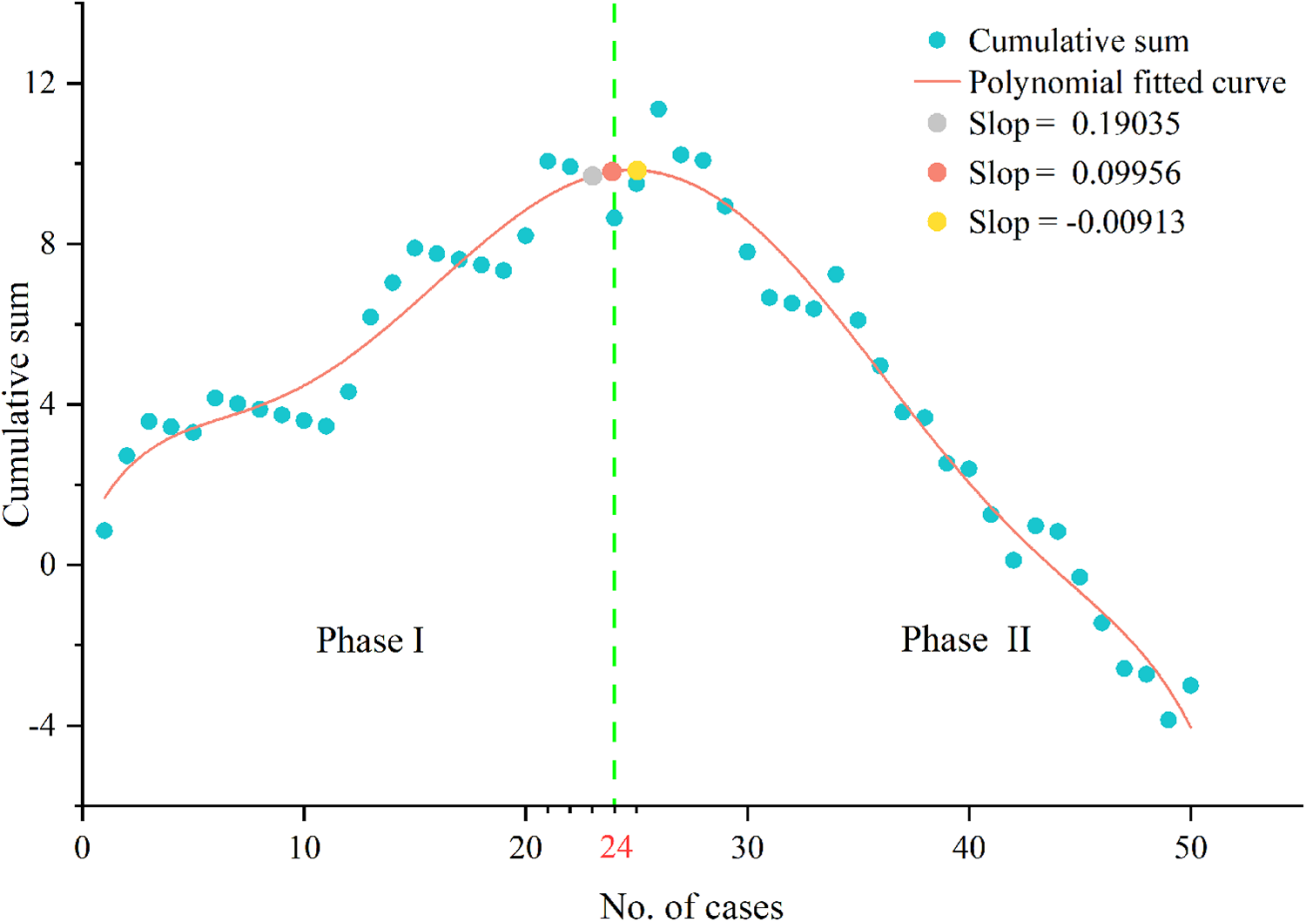
Cumulative sum analysis of surgical competence. The slope of polynomial fitted curve changed from positive to negative when the number of cases over 24.

The CUSUM showed that LC required 24 patients to achieve surgical competence (Figure 4). The polynomial equation for best fit was $y\;=\;0.6722\;+\;1.19891\;\times \;x^{1}\;-\;0.20634x^{2}\;+\;0.01867x^{3}$ $-\;7.44682E^{-4}\;\times \;x^{4}\;+\;1.31087E^{-5}\;\times \;x^{5}\;-\;8.47908E^{-8}\;\times \;x^{6}$,with a r-square value of 0.96315. LC was divided into two distinct phases: phase I (initial 24 cases) and phase II (subsequent 26 cases).

### 3.4. Interphase comparisons between the learning phases

The baseline characteristics and perioperative outcomes of two learning phases are listed in Tables 1,2, respectively. The baseline demographics were comparable between the two phases, including tumor size (3.33 vs. 2.90 cm, *p* = 0.74), RENAL score (5.46 vs. 5.77, *p* = 0.39) and MAP score (34.6% vs*.* 20.9%, *p* = 0.55). The mean OT (133.5 vs*.* 115.31 min; *p* = 0.04), the mean console time (103.21 vs. 81.27 min; *p* = 0.01) and mean PLOS (5.33 vs*.* 4.30 days; *p* = 0.04) were shorter in the phase II as compared to the phase I. However, no statistically significant differences were observed in terms of EBL, eGFR change, Hb change, complications, trifecta achievement rate and pathological outcomes between the two phases (all *p* ≥ 0.05).

## 4. Discussion

The primary goals of PN include control tumors, avoid perioperative complications, and renal functional preservation (18). Compared to RN, PN has demonstrated superior renal functional preservation in patients who underwent surgery for renal tumors (19, 20). It was confirmed that preoperative renal function, quantity of preserved renal parenchyma, and warm ischemia time (WIT) were related to postoperative renal function after PN, among which WIT is an important and modifiable factor that can be modified by adapting surgical technique (21). Currently, the optimal warm ischemia time remains controversial. Some experts recognized 25 min of WIT as the threshold for acute kidney injury (AKI) (22). It has been suggested that limited WIT (≤10 min) had no consequences on renal function (23). Other studies have shown that limited or zero ischemia time might have not a significant impact on renal function (24–26). Nevertheless, Bertolo et al. demonstrated that better postoperative renal functional outcomes for Offc-RAPN compared to Onc-RAPN within 1-year follow-up after surgery (27). According to the study of Flammia et al., main renal artery clamping was significantly associated with chronic kidney disease upstaging (28).Thompson et al. reported that a longer WIT was associated with short-term and long-term renal complications, indicating that every minute of ischemia increases the risk to renal function (4). It is important to note that the severity of renal injury could not be completely reflected by Scr, and renal injury increased with the duration of ischemia, which means that there is no absolutely safe WIT (29). Furthermore, warm ischemia is an independent prognostic factor of ipsilateral parenchymal atrophy and one of the most significant predictors of ipsilateral functional decline after PN (30). Therefore, it is necessary to minimize renal ischemia during PN.

Since White et al. (5) reported the initial implementation of RAPN without renal hilar clamping, Offc-RAPN has been increasingly accepted worldwide (8, 31–33). However, the learning process of Offc-RAPN has rarely been reported, necessitating a comprehensive assessment of the LC to guide surgical training and ensure patient safety during the learning process. Determining the ideal parameters for assessing a surgeon's initial experience with Offc-RAPN is difficult because of the undefined LC in this procedure. While OT is typically considered a crucial parameter, other factors such as PLOS, complications, and EBL also contribute to the procedure's success. Additionally, the CUSUM analysis offers a more effective assessment of data trends by quantifying continuous performance over time (34). Consequently, we performed a CUSUM analysis for LC of purely Offc-RAPN based on the assessment indicators of surgical competence including OT, PLOS, EBL, and complications.

Our study had several interesting findings. First, surgeon with prior experience in routine laparoscopic and robotic surgeries appeared to have a relatively short learning curve (LC) for purely Offc-RAPN, achieving surgical competence after 24 cases. Therefore, the LC was divided into two phases: phase I (first 24 cases) and phase II (next 26 cases). Ferriero et al. (35) compared perioperative outcomes between training and expert groups using propensity score matching and observed that the trained group achieved postoperative outcomes comparable to those of expert group after adequate minimally invasive surgery training. However, their study did not determine the specific number of cases required for training. Our study determined the number of cases required to achieve surgical competence in this procedure, contributing to a better understanding of the learning curve and the development of targeted surgical training programs. It is essential to acknowledge that the surgeon's minimally invasive surgery experience and technical proficiency in instrument usage, tissue handling, and intraoperative coordination of the bedside assistant could influence LC and surgical outcomes. Thus, the number of cases required to achieve competence in Offc-RAPN may vary among surgeons and bedside assistants with diverse minimally invasive surgical backgrounds.

Second, we compared perioperative outcomes between phases I and II. Our analysis revealed that the results of phase I longer phase II in terms of mean PLOS (5.33 vs*.* 4.30 days; *p* = 0.04), mean OT (133.5 vs*.* 115.31 min; *p* = 0.04) and mean console time (103.21 vs*.* 81.27 min; *p* = 0.01). Correspondingly, the LOWESS analysis demonstrated a downward trend in PLOS, OT, and console time. To a similar extent, complications,blood loss and eGFR change did not observed significant correlation with surgical experience,while OT and console time decreased with counterpart, as in previous studies (12, 35). These findings suggest that the accumulation of the surgeon's skill and coordination with the bedside assistant could improve the efficiency of tumor resection, hemostasis, and renal reconstruction, eventually contributing to the early postoperative recovery of patient. However, other perioperative outcomes, such as EBL, Hb change, eGFR change, complications, and trifecta achievement, did not show significant improvements in phase II (all *p *> 0.05). It should be noted that due to the limited research available on defining protocols and outcomes for renal enhanced recovery after surgery (ERAS) protocols for PN (36), all patients underwent PN at our center were subjected to the postoperative management protocols as previously described.

Third, we found that purely Offc-RAPN was safe and feasible even in the initial phase of LC; even in the first 24 cases, the mean EBL was only 117.92 ml, and the mean eGFR change was −3.38%. Of all cases in this phase, only one patient (4.2%) experienced Clavien-Dindo grade ≥2 complications, and no positive surgical margins were observed. Trifecta was observed in most patients (22/24, 91.7%). Hence our results, which are consistent with those of previous reports (7, 37, 38). As highlighted by Bertolo et al. (39), Offc-RAPN can be carried out safely and effectively in the initial experience of this procedure.

A major surgery-related complication of PN is hemorrhage (40), and severe cases may require embolization or reoperation. The Hypotensive anesthesia technique (41) and sequential preplaced suture renorrhaphy technique (42) have been described to minimize intraoperative hemorrhage during RAPN. In our experience, with the maintenance of a clear visual field and an increase of pneumoperitoneum pressure, bipolar electrocautery can effectively control most of intraoperative bleeding. The relatively avascular plane was advanced by blunt dissection reducing parenchymal bleeding. However, it is necessary to consider the possibility of a vessel adjacent to the obstructed areas when repeated obstruction occurs during blunt dissection along the tumor capsule. In a study by Kaczmarek et al. (6), Offc-RAPN had an acceptable and higher mean EBL (228 vs*.* 157 ml, *p* = 0.009) than Onc-RAPN, which is similar to the results of our series (119.2 ml). Therefore, effective intraoperative hemostasis in a state of zero renal ischemia can be achieved using precise operative technique and adept coordination with the assistant.

While representing the initial exploration of the learning process of purely Offc-RAPN, our study has certain limitations that may limit the generalizability of the findings to surgeons of varying experience and center volume (43). First, it was a small sample size retrospective study with the potential confounding bias and observational bias. Furthermore, the selection of patients is crucial for Offc-RAPN. Since our study was conducted by a single surgeon at a single center, our indications for Offc-RAPN may not be generalizable to the entire urologic community. Second, the role of the assistant in the procedure is pivotal, and the impact of assistants on LC during the procedure remains uncertain and requires further investigation. Finally, the assessment of learning curve can be influenced by patient-related factors such as BMI, the degree of APF and the anatomical characteristics of the tumor. The inclusion of some relatively easy cases in the initial cohort of Offc-RAPN also affects the accuracy of learning curve. Therefore, larger scaled and more comprehensive studies are necessary to confirm our conclusion.

## 5. Conclusion

Surgeons with experience in laparoscopic and robotic surgery achieved early proficiency in Offc-RAPN after 24 cases, leading to reductions in OT, console time, and PLOS. Moreover, Offc-RAPN alone is safe and feasible even in the initial phase of LC for an experience surgeon.

## Data Availability

The raw data supporting the conclusions of this article will be made available by the authors, without undue reservation.
